# Why Do People Marry?

**Published:** 1889-11

**Authors:** 


### WHY DO PEOPLE MARRY ?

I have said that in this degenerate age men seldom, if ever, marry for love, while women generally do. Men marry in order to gratify passion or for money, position, or in order to add to personal convenience or comfort.

Sentiment seldom enters into the calculations of a man of mature years and experience, and he that asserts otherwise shows his lack of knowledge of mankind.

Young men, under thirty, frequently do marry for love. Old ones seldom.

These facts show that our social system is all wrong, all astray, for such sentiments and motives always lead to misunderstanding, and frequently to divorce.

No union based upon a lack of sentiment can be permanently happy. Mothers teach their daughters that marriage is their whole occupation, but they discourage any union or engagement not based upon wealth as a consequence.

A close observer once said that "at sixteen a woman marries for love, at twenty for position, at twenty-five for money and at thirty neither money, love nor position enter into the matter, for the question is, 'Where is the man ?' This wretched old cynic saw with jaundiced eyes the world of to-day, and understood society as it is with all its artificiality, hypocrisy and selfishness."

In olden times parents were only too glad to have their daughters marry the man they loved, and money, show and position were secondary considerations. Love, honour and energy counted for much ; now they count for little, and a regrettable state of affairs it is, too.

Money is the God, and money gets the choicest of the sex in marriage. "'Tis true 'tis pity, and pity 'tis 'tis true."

My esteemed friend and critic may doubt what I say, but facts support the truth of it, and he is but a slow man who fails to see the tendency of the age, and seeks to ignore it.

Marriage of to-day is a mere contract in which the man seeks for wealth, position or convenience, and in which the woman, recognising her helpless and dependent condition, accepts the situation, marries for love when she can, but marries for anything else sooner than become an old maid, with all the old maids disadvantages and wretchedness.—*Ex.*

